# High signal intensity on T2 weighted cardiac magnetic resonance imaging in hypertrophic cardiomyopathy: Is it a marker of myocardial injury?

**DOI:** 10.1186/1532-429X-17-S1-P289

**Published:** 2015-02-03

**Authors:** Shi Chen, Luo Yong, Liwei Huang, Jiayu Sun, Tianjing Zhang, Qing Zhang, Yucheng Chen

**Affiliations:** West China Hosptial, Chengdu, China; Department of Cardiovascular Ultrasound and Noninvasive Cardiology, Sichuan Academy of Medical Sciences and Sichuan People’s Hospital, Chengdu, Chengdu, China; MR Collaborations NE Asia, Siemens Healthcare, Beijing, China

## Background

Previous studies observed the phenomenon of high signal intensity on T2 -weighted image of cardiac magnetic resonance imaging (CMR) in hypertrophic cardiomyopathy(HCM). However, the underlying histopathologic mechanism is unclear. Elevated cardiac troponin can be detected in some HCM patients. A reasonable hypothesis is that myocardial T2-high signals is a potential marker of myocaridal injury in HCM. We sought to investigate the association between cardiac troponin and the extent of T2-high signals in HCM patients.

## Methods

Forty-four HCM patients underwent 3.0T CMR scanning. On T2-weighted images, the number of segments with high-signal intensity(myocardium to skeletal muscle signal intensity ratio>2)and the percentage of high-signal area (>2 SD above remote tissue) were measured in 16 myocardial segments along the LV mid-myocardial circumference on 3 short-axis images. The level of high sensitivity cardiac troponin T(hs-cTnT) was also assessed.

## Results

Myocardial T2-high signals were indentified in 33(75%)patients and 144(20.5%) segments.Elevated hs-cTnT was observed in 28(63.6%)patients.Cochran-Armitage test showed a statistically significant trend of increasing level of hs-cTnT with elevating number of segments with myocardial T2-high signal (p=0.002).Then,Pearson^,^s test showed the percentage of myocardium with T2-high signal significantly associated with the hs-cTnT level(r=0.388,P=0.009)

## Conclusions

Myocardial T2-high signals can be considered as a marker of myocardial injury in HCM patients.

**Figure 1:**
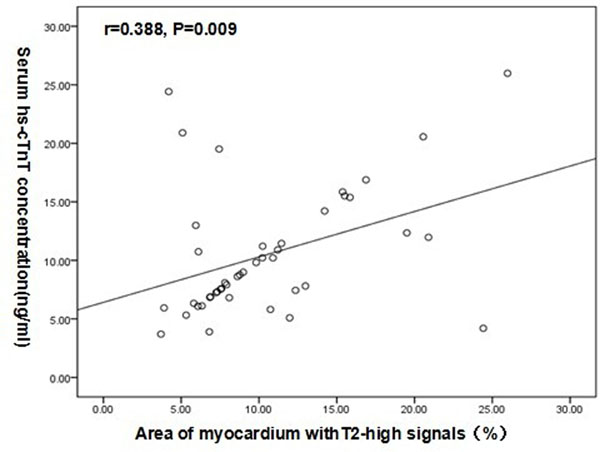
The relationship between the percentage of myocardium with T2-high signal and the level of high sensitivity cardiac troponin T(hs-cTnT)in patients with hypertrophic cardiomyopathy.

**Table 1:** Cardiac magnetic resonance characteristics of patients with HCM

	Study participants (n=44)
LVEDV,(ml)	136.1±31.7
LVESV,(ml)	49.1±15.0
LVEF,(%)	62.7±12.0
LV mass,(g)	155.2±54.5
LV mass index,(g/m2)	94.2±34.8
Segments with T2-high signal, n(%)	144(20.5)
Presence of T2-high signal, (%)	10.5±5.4

LVEDV, left ventricular end diastolic volume; LVESV, left ventricular end systolic volume; LVEF, left ventricular ejection fraction;LV left ventricle

